# Post-Cementation Sensitivity of Zirconia Crowns: A Comparative Clinical Study of Glass Ionomer and Self-Adhesive Resin Cements

**DOI:** 10.4317/jced.64124

**Published:** 2026-06-29

**Authors:** Rajmonda Halili, Sebahate Hamiti Alidema

**Affiliations:** 1 Department of Prosthodontics, AAB College, Prishtina, Kosovo; 2 Department of Prosthodontics, Alma Mater Europaea Campus College Rezonanca, Prishtina, Kosovo

## Abstract

**Background:**

This clinical study evaluated post-cementation sensitivity after zirconia crown cementation using two luting agents: a conventional glass ionomer cement (Fuji I) and a self-adhesive dual-cure resin cement (Calibra Ceram).

**Materials and Methods:**

A total of 40 patients requiring zirconia crown restorations were included in the study and randomly allocated to one of two groups according to the luting cement used. In Group I, zirconia crowns were cemented with conventional glass ionomer cement (Fuji I), whereas in Group II they were cemented with self-adhesive dual-cure resin cement (Calibra Ceram). Post-cementation sensitivity was assessed using a visual analogue scale (VAS) at 2 hours after cementation and after 14 days. Sensitivity was evaluated in response to cold, heat, and biting stimuli. Normality was assessed using the Shapiro-Wilk test. As the data were not normally distributed, intragroup comparisons were performed using the Wilcoxon signed-rank test and intergroup comparisons using the Mann-Whitney U test (p &lt; 0.05).

**Results:**

At both evaluation times, the Fuji I group showed higher sensitivity scores than the Calibra Ceram group for cold, heat, and biting stimuli. Cold and heat sensitivity decreased significantly from baseline to day 14 in both groups. For biting sensitivity, a significant reduction was observed in the Fuji I group, whereas the reduction in the Calibra Ceram group was not statistically significant. Between-group comparisons showed significantly lower sensitivity scores in the Calibra Ceram group for all tested stimuli at both time points.

**Conclusions:**

Within the limitations of this clinical study, zirconia crowns cemented with self-adhesive resin cement showed lower post-cementation sensitivity than those cemented with conventional glass ionomer cement. The type of luting cement may therefore influence early postoperative sensitivity and patient comfort following zirconia crown cementation.

## Introduction

Zirconia-based ceramics are widely used in contemporary fixed prosthodontics due to their high flexural strength, fracture resistance, and favorable biocompatibility. These characteristics allow zirconia restorations to be successfully used in both anterior and posterior regions, contributing to their increasing popularity in restorative dentistry (1 , 2). In addition, the growing aesthetic expectations of patients have further increased the demand for metal-free restorations. The clinical success of zirconia crowns depends not only on the restorative material itself but also on the selection of an appropriate luting cement and cementation protocol (3 , 4). Conventional glass ionomer cements have long been used for crown cementation because of their chemical adhesion to tooth structure, fluoride release, and relatively simple clinical handling (3). By contrast, resin-based cements are often recommended for ceramic restorations due to their improved mechanical properties, enhanced marginal adaptation, and reliable polymerization characteristics (3 , 5). Despite the favorable mechanical properties of zirconia restorations, postoperative pulpal sensitivity remains a clinically relevant complication following crown cementation. This phenomenon has been associated with dentin permeability, hydraulic fluid movement within dentinal tubules, and the biological response of the pulp to restorative procedures (6). Previous clinical studies have suggested that the type of luting cement may influence the incidence and severity of post-cementation sensitivity in vital abutment teeth (7). Therefore, the aim of the present clinical study was to evaluate post-cementation sensitivity following zirconia crown cementation using a conventional glass ionomer cement (Fuji I) and a self-adhesive dual-cure resin cement (Calibra Ceram). The null hypothesis was that the type of luting cement would not influence post-cementation sensitivity following zirconia crown cementation.

## Materials and Methods

This clinical study was conducted at the Department of Prosthodontics, AAB College, Prishtina, Kosovo, after approval by the Ethics Committee (approval no. 073/26). Patients requiring zirconia crown restorations in the maxilla were included. Only patients with vital abutment teeth, confirmed by pulp vitality testing, were enrolled. The selected teeth were free from deep caries, fractures, extensive restorations, and previous endodontic treatment. In addition, the abutment teeth had stable occlusion, adequate opposing dentition, and a healthy periodontium or only mild gingivitis without severe periodontal disease. Patients with severe periodontal disease, uncontrolled systemic conditions, or known hypersensitivity to dental materials were excluded. Individuals who were unable to attend follow-up visits or reliably report postoperative sensitivity were also excluded. After eligibility was confirmed and written informed consent was obtained, participants were randomly allocated to the Fuji I or Calibra Ceram group in a 1:1 ratio. A computer-generated randomization sequence was prepared before recruitment by an investigator not involved in the clinical procedures or outcome assessment. Allocation concealment was maintained using sequentially numbered, opaque, sealed envelopes, which were opened immediately before definitive cementation. The luting cement indicated by the allocation code was then used. Patients and the examiner assessing VAS sensitivity scores were blinded to group allocation, whereas the operator could not be blinded because of the different handling and application protocols of the two luting agents. The abutment teeth were clinically examined, anesthetized, and prepared for zirconia crowns. Occlusal and axial tooth reduction was performed to provide adequate space for zirconia crowns, with a uniform taper and rounded line angles to improve retention and reduce stress concentration. A chamfer finish line was prepared, unsupported enamel and carious tissue were removed, and gingival retraction was performed to expose the margin. After tooth preparation, a two-step putty-wash impression technique (Silagum, DMG) was used to capture the details required for laboratory fabrication. Provisional crowns were then cemented with temporary cement (Cavex). Five days later, the provisional crowns were removed, and the prepared abutment teeth were cleaned, dried, and definitively cemented using one of the two luting agents. For zirconia crowns cemented with conventional glass ionomer cement (GC Fuji I, GC Corporation, Tokyo, Japan), the cement was mixed according to the manufacturer's instructions and applied to the entire internal surface of the zirconia crown before seating. The crown was seated with firm pressure, and excess cement was removed after the initial set. Occlusion was then checked. For zirconia crowns cemented with self-adhesive dual-cure resin cement (Calibra Ceram, Dentsply Sirona, USA), the internal surface of the zirconia crown was treated according to the manufacturer's recommendations. The cement was then applied, and the crown was seated with firm pressure. Initial excess cement was removed, and the restoration was light-cured according to the manufacturer's instructions. Occlusion was verified, and the margins were carefully cleaned to ensure complete seating and optimal adaptation. Post-cementation sensitivity was assessed using a visual analogue scale (VAS) at 2 hours after cementation and at the 14-day follow-up visit. Three stimuli were used: cold, heat, and biting. Cold sensitivity was tested by applying a cotton pellet sprayed with refrigerant spray (Endo-Frost) to the buccal surface of the restored tooth. Heat sensitivity was evaluated by applying heated gutta-percha to the buccal surface of the tooth under controlled conditions. Biting sensitivity was assessed by asking the patient to bite gently on a cotton roll positioned over the restored tooth. After each stimulus, patients rated the intensity of sensitivity on a 10-point VAS, where 0 indicated no sensitivity and 10 indicated the worst imaginable pain. All assessments were performed by the same examiner to ensure consistency. Statistical analysis Statistical analysis was performed using IBM SPSS Statistics software (Version 26, IBM Corp., Armonk, NY, USA). Descriptive statistics were expressed as median and interquartile range (IQR). Normality of distribution was assessed using the Shapiro-Wilk test, which showed that sensitivity scores were not normally distributed. Therefore, non-parametric tests were used. Intragroup comparisons between baseline (2 hours after cementation) and day 14 were performed using the Wilcoxon signed-rank test, whereas intergroup comparisons at each time point were performed using the Mann-Whitney U test. A p value &lt; 0.05 was considered statistically significant.

## Results

**Figure 1:**
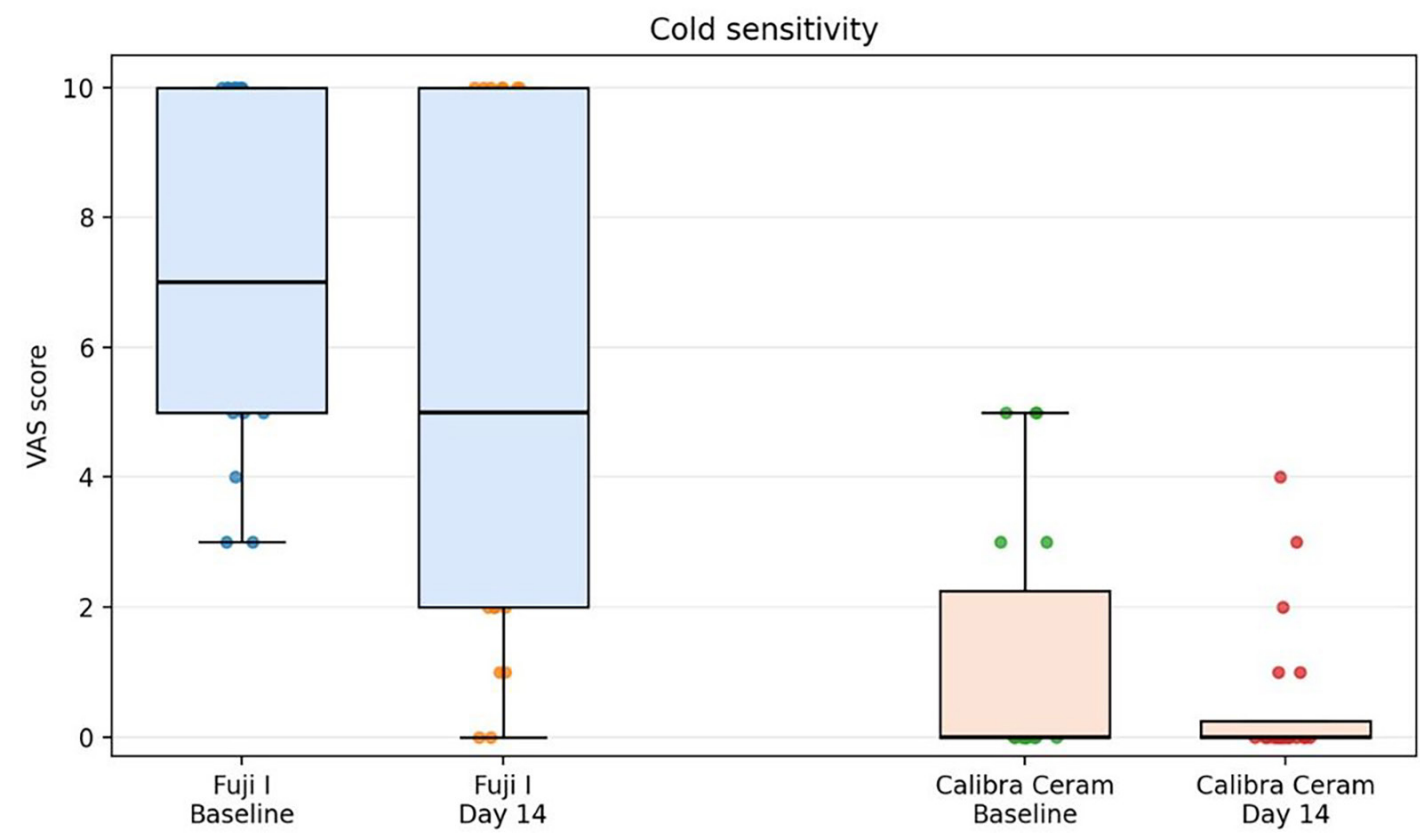
Distribution of VAS scores for cold sensitivity at baseline and after 14 days in the Fuji I and Calibra Ceram groups.

**Figure 2:**
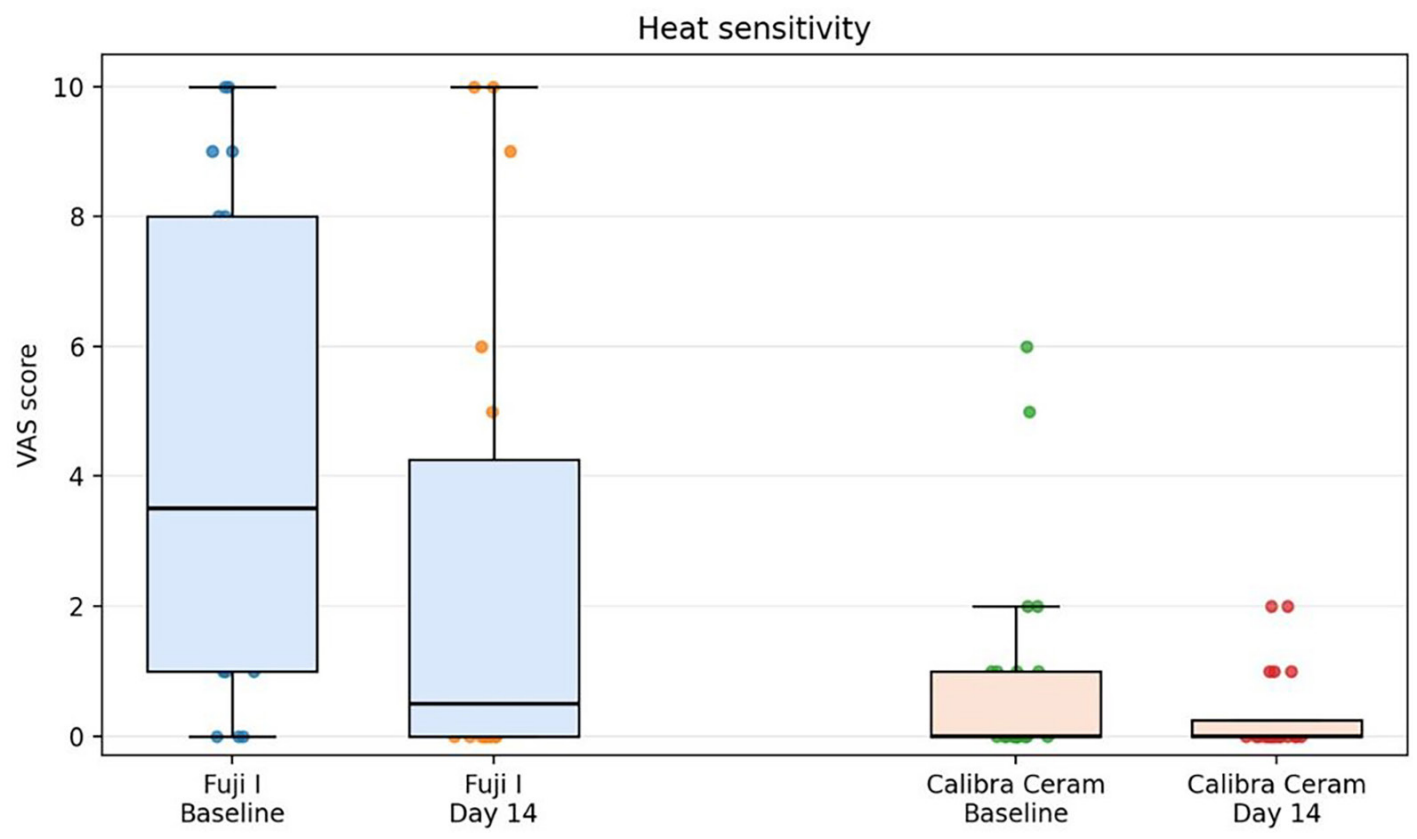
Distribution of VAS scores for heat sensitivity at baseline and after 14 days in the Fuji I and Calibra Ceram groups.

**Figure 3:**
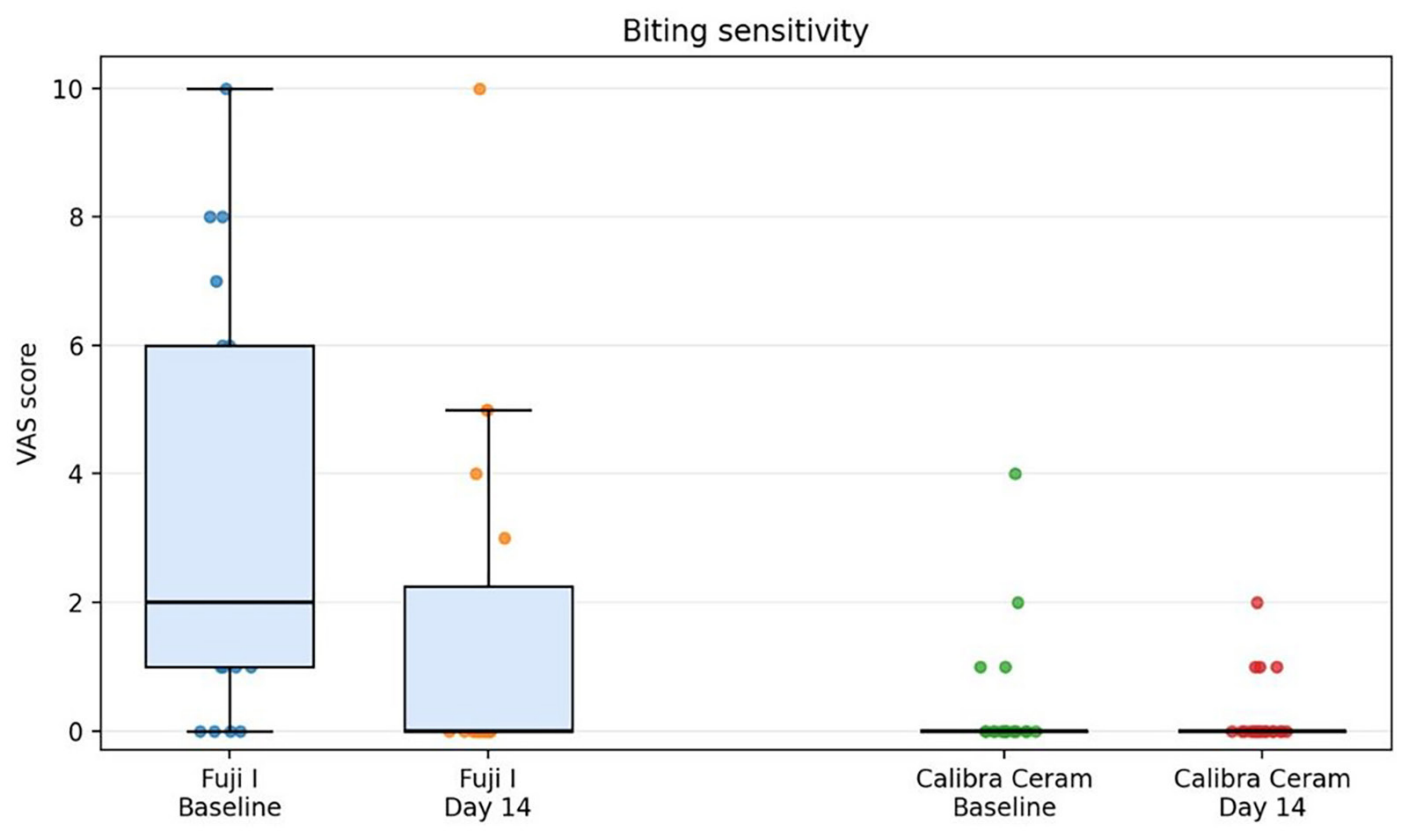
Distribution of VAS scores for biting sensitivity at baseline and after 14 days in the Fuji I and Calibra Ceram groups.

A total of 40 patients were included in the study and randomly allocated into two equal groups according to the computer-generated allocation sequence. Group I received zirconia crowns cemented with conventional glass ionomer cement (Fuji I), whereas Group II received crowns cemented with the self-adhesive dual-cure resin cement Calibra Ceram. Table 1 and Figures 1-3 present the non-parametric distribution of post-cementation sensitivity scores at baseline (2 hours after cementation) and after 14 days.

Table 1

1

2

3

Overall, the Fuji I group showed higher VAS scores than the Calibra Ceram group for cold, heat, and biting stimuli at both evaluation times. For cold sensitivity, the Fuji I group showed a median VAS score of 7.0 (IQR 5.0-10.0) at baseline, which decreased to 5.0 (IQR 2.0-10.0) after 14 days (p = 0.0018). In the Calibra Ceram group, the median score decreased from 0.0 (IQR 0.0-2.2) at baseline to 0.0 (IQR 0.0-0.2) after 14 days (p = 0.0059). Between-group comparisons showed significantly lower cold sensitivity in the Calibra Ceram group both at baseline and at day 14 (p &lt; 0.001 for both comparisons). For heat sensitivity, the Fuji I group showed a median score of 3.5 (IQR 1.0-8.0) at baseline and 0.5 (IQR 0.0-4.2) after 14 days, representing a significant reduction over time (p = 0.0014). In the Calibra Ceram group, heat sensitivity decreased from 0.0 (IQR 0.0-1.0) at baseline to 0.0 (IQR 0.0-0.2) after 14 days (p = 0.0412). Between-group analysis again demonstrated significantly lower sensitivity in the Calibra Ceram group at baseline (p &lt; 0.001) and at day 14 (p = 0.0316). For biting sensitivity, the Fuji I group showed a median score of 2.0 (IQR 1.0-6.0) at baseline, which decreased to 0.0 (IQR 0.0-2.2) after 14 days (p = 0.0069). In the Calibra Ceram group, the median biting sensitivity remained 0.0 (IQR 0.0-0.0) at both time points, and the reduction was not statistically significant (p = 0.1797). Nevertheless, between-group comparisons still showed significantly lower biting sensitivity in the Calibra Ceram group both at baseline (p &lt; 0.001) and after 14 days (p = 0.0453). Overall, sensitivity scores decreased over time in both groups, with the most consistent reductions observed for thermal stimuli. Across all tested stimuli and both evaluation times, lower sensitivity values were consistently observed in the Calibra Ceram group.

## Discussion

The present clinical study evaluated post-cementation sensitivity following zirconia crown cementation using two different luting agents. Lower sensitivity scores were observed in zirconia crowns cemented with the self-adhesive resin cement Calibra Ceram compared with those cemented with conventional glass ionomer cement (Fuji I). In addition, sensitivity decreased during the follow-up period in both groups, particularly for thermal stimuli. These findings indicate that the type of luting cement may influence post-cementation sensitivity, leading to rejection of the null hypothesis. Cold sensitivity showed the highest VAS scores among the evaluated stimuli, especially in the Fuji I group at baseline. These findings are consistent with previous reports indicating that thermal stimulation, particularly cold, represents the most common trigger of postoperative sensitivity after indirect restorations (6). However, the findings of another study (7) reported no significant difference between glass ionomer cement and resin cement in vital teeth with respect to post-cementation hypersensitivity. This inconsistency may be explained by variations in the chemical composition and individual characteristics of the resin-based cements used. Furthermore, Shenoy et al. (6) noted that a certain degree of pulpal trauma is inevitable during tooth preparation due to the sectioning of dentinal tubules, making complete prevention of sensitivity unlikely. In this context, self-adhesive resin cement may offer an advantage because it interacts with the smear layer without completely removing it. This modified interaction may lessen pulpal irritation and, consequently, reduce post-cementation hypersensitivity (8). The lower sensitivity scores found in the Calibra Ceram group may be related to the physicochemical properties of resin-based cements. These cements generally provide better marginal adaptation and sealing, which may help reduce microleakage and restrict dentinal fluid movement within the tubules (3 , 9 , 10). As a result, pulpal irritation may be reduced, leading to lower postoperative sensitivity. This effect may also be linked to the better adhesive properties of resin cement and its closer adaptation to tooth structure. By reducing dentin permeability, self-adhesive resin cements may limit thermal and mechanical stimulation of the pulp. This explanation is supported by Chang et al. (11)., who reported lower microleakage around zirconia crown margins with self-adhesive resin cement than with resin-modified glass ionomer cement. In addition, Kimyai et al. (12) emphasized the role of dentin sealing and bond stability in the performance of self-adhesive resin cement, whereas Kaviani and Khansari Nejad (13). showed that microleakage is influenced by factors related to the tooth-cement interface. Overall, these findings suggest that the improved sealing ability of resin cement may have contributed to the lower postoperative sensitivity observed in our study. By comparison, glass ionomer-based cements may allow greater early microleakage, which may help explain the higher sensitivity scores recorded in that group (11). Another important finding of the present study was the reduction in sensitivity scores after 14 days in both groups. Similar trends have been reported in previous clinical studies evaluating postoperative sensitivity following crown cementation (7 , 14 , 15). The gradual reduction in symptoms may be explained by pulpal recovery and the stabilization of the tooth-restoration interface over time. From a clinical perspective, the findings of the present study suggest that the choice of luting cement may have a direct influence on early patient comfort after zirconia crown cementation. Although conventional glass ionomer cement remains widely used because of its ease of manipulation, fluoride release, and long clinical history, the higher sensitivity scores observed in the Fuji I group indicate that clinicians should carefully evaluate its use in vital abutment teeth, particularly in patients with deep preparations, thin remaining dentin, or a previous history of dental hypersensitivity. In such clinical situations, self-adhesive resin cement may represent a more favorable option because it may provide improved marginal sealing and reduced dentin permeability, thereby limiting thermal and mechanical stimulation of the pulp (3 , 4 , 16). The lower sensitivity values observed in the Calibra Ceram group may be clinically relevant, particularly during the early postoperative period, when patient comfort can strongly influence treatment satisfaction. Even when transient, post-cementation sensitivity may cause patient concern and lead to additional clinical visits. Therefore, the choice of luting cement should not be based only on retention, mechanical strength, or ease of use, but should also consider its potential effect on pulpal response and short-term postoperative comfort. These findings support an individualized cementation strategy that takes into account tooth vitality, preparation depth, dentin exposure, occlusal conditions, moisture control, and the characteristics of the restorative material. However, the relatively small sample size may limit the generalizability of the results, and the 14-day follow-up period allows assessment only of early post-cementation sensitivity, not long-term pulpal response, marginal integrity, restoration survival, or late complications. In addition, sensitivity was assessed using patient-reported VAS scores, which, although clinically practical and widely used, remain subjective and may be influenced by individual pain thresholds, anxiety, previous dental experiences, and interpretation of discomfort. Despite these limitations, the present findings provide clinically useful evidence that self-adhesive resin cement may be associated with lower early post-cementation sensitivity in vital teeth restored with zirconia crowns. This may support more biologically informed cement selection, particularly in cases where postoperative comfort and pulpal protection are important clinical considerations.

## Conclusions

Within the limitations of this clinical study, zirconia crowns cemented with the self-adhesive dual-cure resin cement (Calibra Ceram) showed lower post-cementation sensitivity than those cemented with conventional glass ionomer cement (Fuji I). Sensitivity to cold, heat, and biting stimuli decreased over time in both groups, with the greatest reduction observed for thermal stimuli. These findings suggest that the type of luting cement may influence early postoperative pulpal response and patient comfort after zirconia crown cementation. Self-adhesive resin cements may therefore be associated with a more favorable short-term clinical outcome in terms of postoperative sensitivity in vital abutment teeth. Further clinical studies with larger sample sizes and longer follow-up periods are needed to confirm these findings and to clarify the long-term influence of luting cements on postoperative sensitivity associated with zirconia restorations.

## Figures and Tables

**Table 1:** Median (IQR) VAS scores for cold, heat, and biting sensitivity at baseline (2 hours) and after 14 days, with within-group and between-group comparisons.

Stimulus	Group	Baseline,median (IQR)	14 days,median (IQR)	Within-groupp value	Between-groupp at baseline	Between-groupp at 14 days
Cold	Fuji I	7.0 (5.0-10.0)	5.0 (2.0-10.0)	0.0018	<0.001	<0.001
	Calibra Ceram	0.0 (0.0-2.2)	0.0 (0.0-0.2)	0.0059		
Heat	Fuji I	3.5 (1.0-8.0)	0.5 (0.0-4.2)	0.0014	<0.001	0.0316
	Calibra Ceram	0.0 (0.0-1.0)	0.0 (0.0-0.2)	0.0412		
Biting	Fuji I	2.0 (1.0-6.0)	0.0 (0.0-2.2)	0.0069	<0.001	0.0453
	Calibra Ceram	0.0 (0.0-0.0)	0.0 (0.0-0.0)	0.1797		

VAS, visual analogue scale; IQR, interquartile range. Intragroup comparisons were performed using the Wilcoxon signed-rank test. Between-group comparisons were performed using the Mann-Whitney U test. A p value < 0.05 was considered statistically significant.

## Data Availability

The datasets used and/or analyzed during the current study are available from the corresponding author.
